# Agapanthussaponin A from the Underground Parts of *Agapanthus africanus* Induces Apoptosis and Ferroptosis in Human Small-Cell Lung Cancer Cells

**DOI:** 10.3390/molecules30153189

**Published:** 2025-07-30

**Authors:** Tomoki Iguchi, Tamami Shimazaki, Yoshihiro Mimaki

**Affiliations:** Department of Medicinal Pharmacognosy, School of Pharmacy, Tokyo University of Pharmacy and Life Sciences, Tokyo 192-0392, Japan; y184116@toyaku.ac.jp

**Keywords:** *Agapanthus africanus*, agapanthussaponin A, SBC-3 cell, apoptosis, ferroptosis, steroidal glycoside

## Abstract

To explore the potential seed compounds from natural products as anticancer agents against small-cell lung cancer (SCLC), the underground parts of *Agapanthus africanus*, a plant commonly used for ornamental purposes, were investigated. Three spirostan-type steroidal glycosides (**1**–**3**) were isolated and identified by nuclear magnetic resonance spectral analysis. Compounds **1**–**3** exhibited cytotoxicity against SBC-3 human SCLC cells, with IC_50_ values of 0.56, 1.4, and 7.4 µM, respectively. Compound **1**, also known an agapanthussaponin A, demonstrated the most potent cytotoxicity among the isolated compounds and was evaluated for its apoptosis- and ferroptosis-inducing activities. Compound **1** arrested the cell cycle of SBC-3 cells in the G_2_/M phase and induced apoptosis primarily via the mitochondrial pathway, characterized by caspases-3 and -9 activation, loss of mitochondrial membrane potential, and overproduction of reactive oxygen species. Additionally, **1** triggered ferroptosis via a dual mechanism consisting of enhanced cellular iron uptake through upregulation of transferrin and transferrin receptor 1 expression and impaired glutathione synthesis via downregulation of both xCT and glutathione peroxidase 4 expression. Compound **1** induces cell death via the apoptosis and ferroptosis pathways, suggesting its promise as a seed compound for the development of anticancer therapeutics against SCLC.

## 1. Introduction

Cancer remains a critical global health challenge that exerts a substantial burden on healthcare systems worldwide. According to the Global Cancer Observatory of the Health, an estimated 20 million new cancer cases and 9.7 million cancer-related deaths were reported globally in 2022 [1]. Among the diverse array of malignancies, lung cancer is particularly notable, exhibiting both high incidence and mortality rates compared to other cancer types [1]. More specifically, in the United States, lung cancer ranks as the leading cause of cancer-related mortality in both sexes, accounting for approximately 20% of all cancer-related deaths in 2021 [2]. Lung cancer is classified into two major histological subtypes: non-small cell lung cancer (NSCLC) and small cell lung cancer (SCLC). SCLC is among the most aggressive malignancies, accounting for approximately 13–15% of all lung cancer cases [3,4]. The exceptionally poor prognosis of this disease is evidenced by dismal clinical outcomes, with a median overall survival (OS) of less than 12 months and 5-year OS rates not exceeding 7% [3,5]. Since the 1980s, platinum-based chemotherapy has served as the cornerstone of first-line treatment for SCLC [5]. In recent years, the integration of immune checkpoint inhibitors with conventional chemotherapy has been explored as a strategy to enhance therapeutic outcomes in SCLC [6]. However, effective treatment options for relapsed SCLC remain limited, highlighting the urgent need for novel anticancer agents.

As part of our ongoing phytochemical investigations of higher plants aimed at the exploration of potential seed compounds for novel anticancer agents targeting SCLC, we have isolated cytotoxic triterpene glycosides from *Saponaria officinalis* seeds [7], phenolic derivatives from the underground parts of *Eremurus robustus* [8], and steroidal glycosides from *Allium cristophii*×*A. macleanii* ‘Globemaster’ bulbs [9], from *A. atropurpureum* bulbs [10], and from *Ornithogalum thyrsoides* bulbs [11], as well as bufadienolides from the whole plants of *Helleborus niger* [12]. In our previous study, we reported the isolation of 16 novel steroidal glycosides from the highly polar fraction of the extract obtained from the underground parts of *Agapanthus africanus* along with their cytotoxicity against SBC-3 human SCLC cells [13]. In addition, the previous study revealed that one of the isolated compounds from the underground parts of *A. africanus* induces apoptosis in SBC-3 cells through a mitochondria-mediated pathway, accompanied by the overproduction of reactive oxygen species (ROS) [13].

Since ferroptosis [14] induction is a promising therapeutic strategy for cancer treatment, ferroptosis-inducing compounds have been extensively investigated. Ferroptosis, an iron-dependent form of programmed cell death, is distinct from apoptosis and is characterized by excessive iron accumulation in the cytoplasm. This accumulation triggers an iron-driven Fenton reaction, leading to ROS generation and lipid peroxidation [15]. Previously, the ferroptosis-inducing activity of major triterpene and steroid saponins, such as timosaponin AIII [16], albiziabioside A [17], polyphyllin III [18], dioscin [19], formosanin C [20], and paris saponin VII [21], has been reported; however, the ferroptosis-inducing activity of saponins in SCLC has not been adequately investigated. Therefore, in the present study, we conducted a phytochemical investigation of the underground parts of *A. africanus*, including the isolation and structural determination of three steroidal glycosides (**1**–**3**). Compounds **1**–**3**, designated as agapanthussaponins A–C, were previously isolated and structurally characterized from *A. inapertus* [22]. The cytotoxicity of **1**–**3** against SBC-3 cells was subsequently evaluated. Furthermore, **1** was investigated for its potential to induce apoptosis and ferroptosis in SBC-3 cells.

## 2. Results and Discussion

### 2.1. Structure Identification

**Figure 1:**
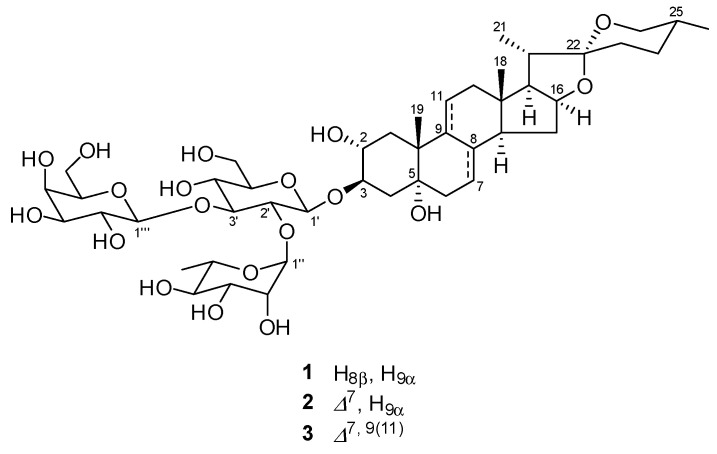
Chemical structures of **1**–**3**.

Prior to phytochemical investigation, the cytotoxicity of the methanol (MeOH) extract of the underground parts of *A. africanus* and the MeOH eluted fraction of the MeOH extract was evaluated against SBC-3 cells. The MeOH extract and the MeOH eluted fraction showed cytotoxicity with IC_50_ values of 3.7 ± 0.033 μg/mL and 0.66 ± 0.0067 μg/mL, respectively. Subsequently, the MeOH eluted fraction was subjected to phytochemical investigation using silica gel and octadecylsilanized (ODS) silica gel column chromatography (CC), yielding **1**–**3**. Compounds **1**–**3** were identified mainly through nuclear magnetic resonance (NMR) analysis. Compounds identified from the ^1^H and ^13^C NMR spectroscopic data are as follows: (25*R*)-2α,5α-dihydroxyspirostan-3β-yl *O*-β-d-galactopyranosyl-(1→3)-*O*-[α-L-rhamnopyranosyl-(1→2)]-β-d-glucopyranoside (**1**) [22], (25*R*)-2α,5α-dihydroxyspirostan-7-en-3β-yl *O*-β-d-galactopyranosyl-(1→3)-*O*-[α-L-rhamnopyranosyl-(1→2)]-β-d-glucopyranoside (**2**) [22], and (25*R*)-2α,5α-dihydroxyspirostan-7,9-dien-3β-yl *O*-β-d-galactopyranosyl-(1→3)-*O*-[α-L-rhamnopyranosyl-(1→2)]-β-d-glucopyranoside (**3**) [22], respectively (Figure 1).

### 2.2. Cytotoxicity of ***1***–***3***

**Figure 2:**
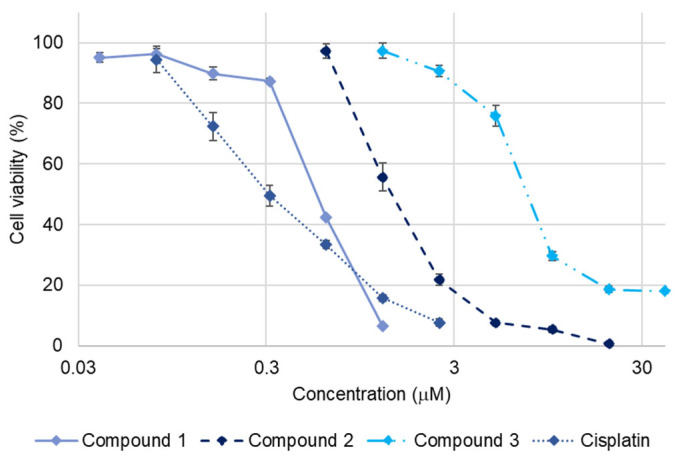
Dose-response curves of cisplatin and **1**–**3** against SBC-3 cells. SBC-3 cells were treated with either cisplatin as a positive control or **1**–**3** for 72 h. The cell viability was evaluated using the MTT assay. Data are presented as mean ± standard error of the mean (SEM) from three independent experiments performed in triplicate.

The cytotoxic activity of **1**–**3** against SBC-3 cells was assessed by the 3-(4,5-dimethylthiazol-2-yl)-2,5-diphenyl-2*H*-tetrazolium bromide (MTT) assay, wherein **1**–**3** exhibited dose-dependent cytotoxicity with IC_50_ values of 0.56 ± 0.0053, 1.4 ± 0.11, and 7.4 ± 0.10 µM, respectively (Figure 2). Comparative analysis of the cytotoxicity of **1**–**3** demonstrated that their cytotoxicity diminished considerably with an increase in the number of double bonds in the structure. The observed decrease in cytotoxicity with increasing number of double bonds in **1**–**3** is consistent with previous structure-activity relationship studies. A similar tendency has been reported for furostan-type steroidal glycosides isolated from the underground parts of *A. africanus*, where compounds with fewer double bonds exhibited greater cytotoxic activity than their more unsaturated analogs [13].

### 2.3. Apoptosis-Inducing Activity of **1**

**Figure 3:**
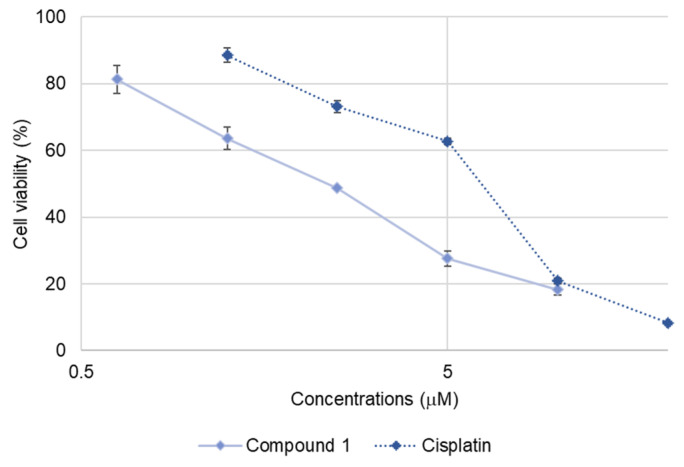
Dose-response curves of cisplatin and **1** against SBC-3 cells. SBC-3 cells were treated with either cisplatin or **1** for 24 h. The cell viability was determined using the MTT assay. Data are presented as mean ± SEM from three independent experiments performed in triplicate.

Compound **1** exhibited relatively potent cytotoxicity against SBC-3 cells, prompting further investigation of its apoptosis-inducing activity in these cells. The IC_50_ value of **1** against SBC-3 cells was determined to be 2.3 ± 0.033 µM based on the dose-response curve generated after 24 h treatment, which helped to optimize the concentration required for subsequent apoptosis assays (Figure 3). Therefore, the apoptosis-inducing activity of **1** was investigated at 2.5 µM, corresponding to its approximate IC_50_ value.

**Figure 4:**
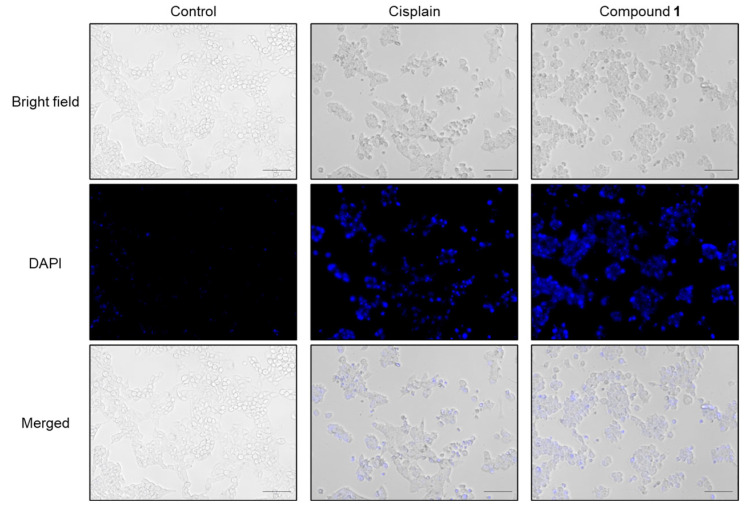
Morphology of SBC-3 cells treated with cisplatin or **1**. SBC-3 cells were treated with either 6 µM of cisplatin or 2.5 µM of **1** for 24 h. Then, the cells were stained with DAPI and observed using a fluorescence microscope. The scale bars indicate 100 µm.

To begin, the morphological changes in the SBC-3 cells following treatment with **1** were examined. SBC-3 cells were treated with either 6 μM of cisplatin or 2.5 µM of **1** for 24 h. After treatment, the cells were stained with 4′,6′-diamidino-2-phenylindole (DAPI) and visualized by fluorescence microscopy. The results revealed increased blue fluorescence intensity and characteristic morphological changes associated with apoptotic cell death, including chromatin condensation and cell shrinkage, in cells treated with **1** compared to the vehicle control (Figure 4).

**Figure 5:**
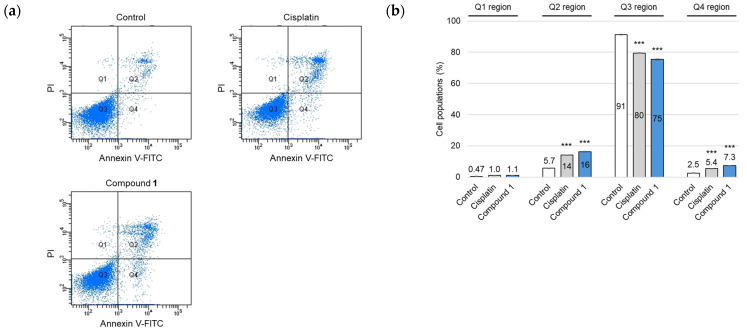
Apoptosis detection in SBC-3 cells followed by the treatment with cisplatin or **1**. (**a**) SBC-3 cells were treated with either 6 μM of cisplatin or 2.5 µM of **1** for 24 h. After treatment, the cells were stained with Annexin V-FITC and PI and analyzed by a flow cytometer. (**b**) The cell population percentages were categorized as follows: dead cells (Q1 region), late apoptotic cells (Q2 region), live cells (Q3 region), and early apoptotic cells (Q4 region). Data are presented as the mean ± SEM from experiments performed in triplicate (*** *p* < 0.001 vs. the corresponding regions of the control).

During the early stages of apoptotic cell death, a notable hallmark is the loss of plasma membrane phospholipid symmetry [23]. This alteration causes phosphatidylserine (PS) molecules, normally confined to the cytoplasmic face, to migrate and become exposed to the external surface of the plasma membrane, enabling PS to bind to Annexin V-fluorescein isothiocyanate (FITC). SBC-3 cells were treated with either 6 µM of cisplatin or 2.5 µM of **1** for 24 h, after which Annexin V-FITC/propidium iodide (PI) double staining and flow cytometry analysis were performed (Figure 5). The flow cytometry scatter plots revealed that treatment with **1** resulted in a significant increase in both early-phase (7.3%; Q4 region) and late-phase (16%; Q2 region) apoptotic cells compared to the control (2.5% and 5.7%, respectively).

**Figure 6:**
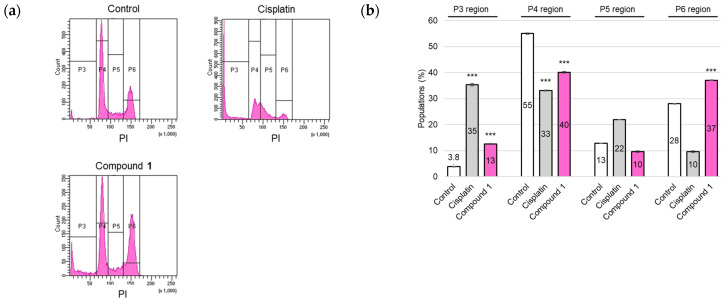
Cell cycle modulation in SBC-3 cells following treatment with cisplatin or **1**. (**a**) SBC-3 cells were treated with either 6 µM of cisplatin or 2.5 µM of **1** for 24 h. After treatment, the cells were fixed, subjected to PI staining, and subsequently analyzed by a flow cytometer. (**b**) The percentages of cells in the sub-G_1_ (P3 region), G_0_/G_1_ (P4 region), S (P5 region), and G_2_/M (P6 region) phases are presented as the mean ± SEM from experiments performed in triplicate (*** *p* < 0.001 vs. the corresponding regions of the control).

The cell cycle distribution of SBC-3 cells after treatment with **1** was evaluated. SBC-3 cells were treated with either 6 µM of cisplatin or 2.5 µM of **1** for 24 h. Fixed cells were stained with PI and subjected to flow cytometric analysis. Flow cytometry histograms revealed a significant increase in G_2_/M phase cell populations (P6 region) in the case of **1** compared to vehicle control, concomitant with a substantial accumulation of the sub-G_1_ peak (P3 region). This result indicates apoptotic cell death (Figure 6). These experimental results indicated that **1** induced cell cycle arrest at the G_2_/M phase and apoptosis in SBC-3 cells.

**Figure 7:**
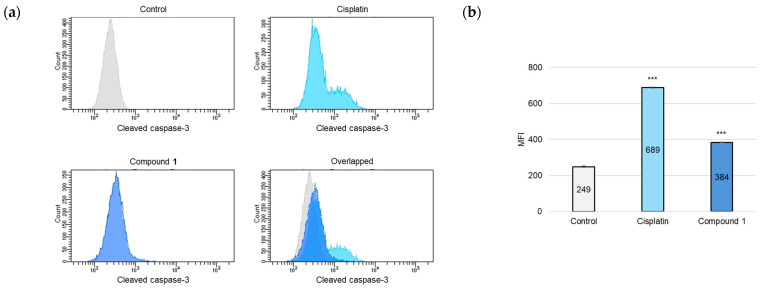

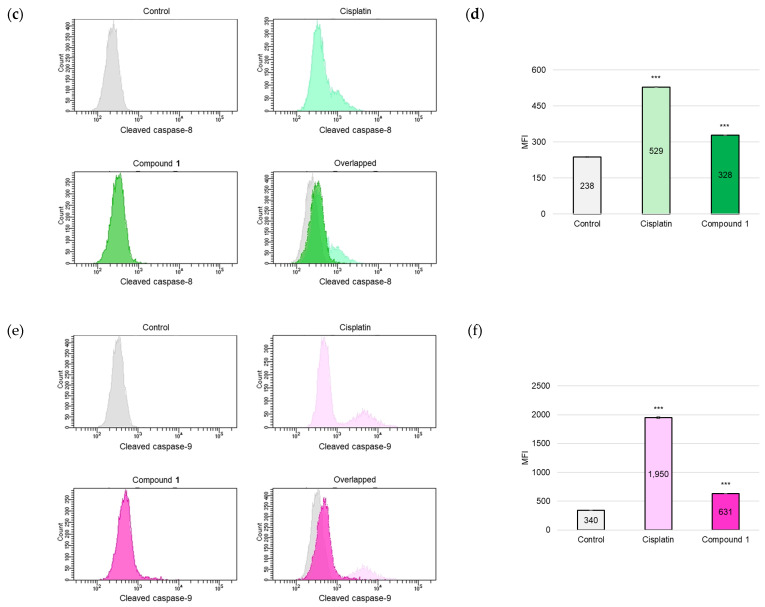
Detection of cleaved caspases-3, -8, and -9 in SBC-3 cells. (**a**,**c**,**e**) SBC-3 cells were treated with either 6 µM of cisplatin or 2.5 µM of **1** for 24 h. Following treatment, the cells were incubated with anti-cleaved caspases-3 (**a**), -8 (**c**), and -9 (**e**), respectively. The antibody-conjugated cells were analyzed by a flow cytometer. (**b**,**d**,**f**) The MFI values are presented as the mean ± SEM from experiments performed in triplicate [(**b**): cleaved caspase-3; (**d**): cleaved caspase-8; and (**f**): cleaved caspase-9 *** *p* < 0.001 vs. the control].

As the activation of cysteine-dependent aspartate-directed proteases (caspases) is a key indicator of apoptotic processes [24], an analysis was conducted to detect the presence of the cleaved forms of caspase-3, caspase-8, and caspase-9. SBC-3 cells were treated with either 6 μM of cisplatin or 2.5 µM of **1**. After 24 h of treatment, the cells were immunostained with anti-cleaved caspases-3, -8, and -9 antibodies and subsequently analyzed by flow cytometry. The mean fluorescence intensity (MFI) values were significantly increased in the cells treated with **1** compared to the vehicle control for cleaved caspases-3, -8, and -9, respectively (Figure 7).

**Figure 8:**
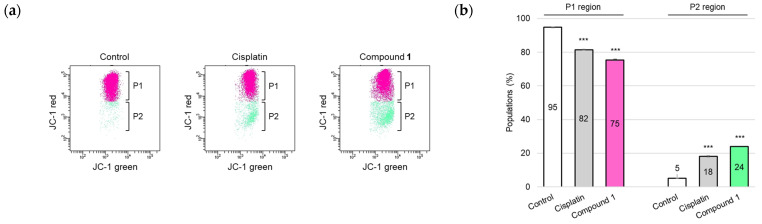
Detection of mitochondrial membrane potential in SBC-3 cells. (**a**) SBC-3 cells were treated with either 6 μM of cisplatin or 2.5 μM of **1** for 24 h. Following treatment, the cells were stained with JC-1 and analyzed by a flow cytometer. (**b**) The percentages of cells in the P1 and P2 regions are presented as the mean ± SEM from experiments performed in triplicate (*** *p* < 0.001 vs. the corresponding regions of the control).

One of the major apoptosis-inducing signaling pathways is the mitochondria-mediated pathway, which is generally referred to as the intrinsic pathway. This pathway involves caspase-9 activation [25], which subsequently activates the downstream executioner caspase, caspase-3. Due to the detection of activation of caspases-3 and -9 in SBC-3 cells treated with **1**, mitochondrial membrane potential (MMP) was evaluated. MMP plays a critical role in facilitating energy conservation during the cellular process of oxidative phosphorylation and maintenance of mitochondrial homeostasis [26]; however, MMP is diminished when mitochondrial dysfunction occurs, particularly during apoptosis. In this study, the mitochondrial condition was assessed using a 5,5′,6,6′-tetrachloro-1,1′,3,3′-tetraethylbenzimidazolylcarbocyanine iodide (JC-1) staining assay. JC-1, a cationic fluorescent dye, exhibits dual emission characteristics: (i) JC-1 aggregates emit red fluorescence with a peak at approximately 590 nm when accumulated in healthy mitochondria with elevated membrane potential, and (ii) JC-1 monomers emit green fluorescence with a maximum at approximately 529 nm when present in depolarized mitochondria characterized by reduced membrane potential [27]. SBC-3 cells were treated with either 6 μM of cisplatin or 2.5 µM of **1** for 24 h. After treatment termination, the cells were stained with JC-1 and analyzed by flow cytometry. The population of cells with depolarized MMP was significantly increased in SBC-3 cells incubated with **1** compared to that in the control (Figure 8).

**Figure 9:**
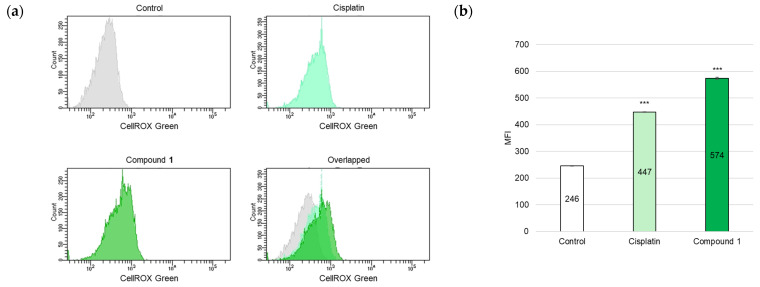
Detection of ROS in SBC-3 cells. (**a**) SBC-3 cells were treated with either 6 μM of cisplatin or 2.5 μM of **1** for 24 h. Following treatment, the cells were stained with CellROX Green and analyzed by a flow cytometer. (**b**) The MFI values are presented as the mean ± SEM from experiments performed in triplicate (*** *p* < 0.001 vs. the control).

ROS play crucial roles in various organisms and participate in apoptotic cell death. Moreover, mitochondria represent a primary ROS-producing cellular organelle [28]. Based on the observed decrease in the MMP of SBC-3 cells treated with **1**, ROS were detected. SBC-3 cells were treated with either 6 µM of cisplatin or 2.5 µM of **1** for 24 h. After treatment, the cells were stained with CellROX Green and analyzed by flow cytometry. The MFI value significantly increased in cells treated with **1** compared to that in the vehicle control (Figure 9).

These comprehensive results demonstrated that **1** induces apoptosis in SBC-3 cells primarily through the mitochondrial pathway, as evidenced by caspases-3 and -9 activation, mitochondrial membrane potential loss, and ROS overproduction, concomitant with cell cycle arrest at the G_2_/M phase.

### 2.4. Ferroptosis-Inducing Activity of **1**

**Figure 10:**
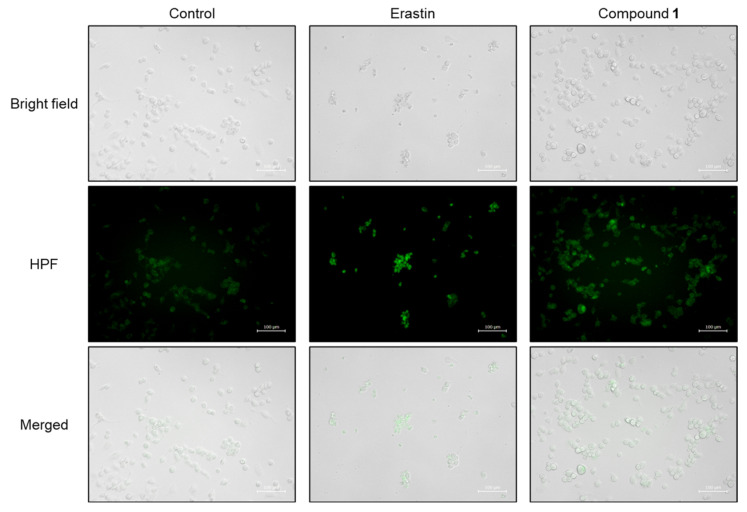
Detection of hydroxyl radicals in SBC-3 cells. SBC-3 cells were treated with either 0.5 µM of erastin or 2.5 µM of **1** for 24 h. Then, the cells were stained with HPF and observed using a fluorescence microscope. The scale bars indicate 100 µm.

ROS play crucial roles in both apoptosis and ferroptosis [29]. As ROS overproduction was observed in SBC-3 cells treated with **1**, it was hypothesized that ferroptosis might be induced in SBC-3 cells treated with **1**. The major characteristics of ferroptosis are accumulation of cellular Fe^2+^ and lipid peroxidation. Hydroxyl radicals are generated from hydrogen peroxide (H_2_O_2_) via the Fenton reaction, in which iron catalyzes the conversion of H_2_O_2_ into highly reactive hydroxyl radicals. These hydroxyl radicals are recognized as initiators of lipid peroxidation, and when overproduced, they play a vital role in this process [30]. Initially, hydroxyl radicals were detected in SBC-3 cells treated with **1**. SBC-3 cells were treated with either 0.5 µM of erastin, a ferroptosis inducer [31], or 2.5 μM of **1** for 24 h. After treatment, the cells were stained with hydroxyphenyl fluorescein (HPF), a hydroxyl radical detection reagent, and monitored using a fluorescence microscope. Enhanced green fluorescent emission derived from hydroxyl radicals was observed in the cells treated with **1** compared to the control (Figure 10).

**Figure 11:**
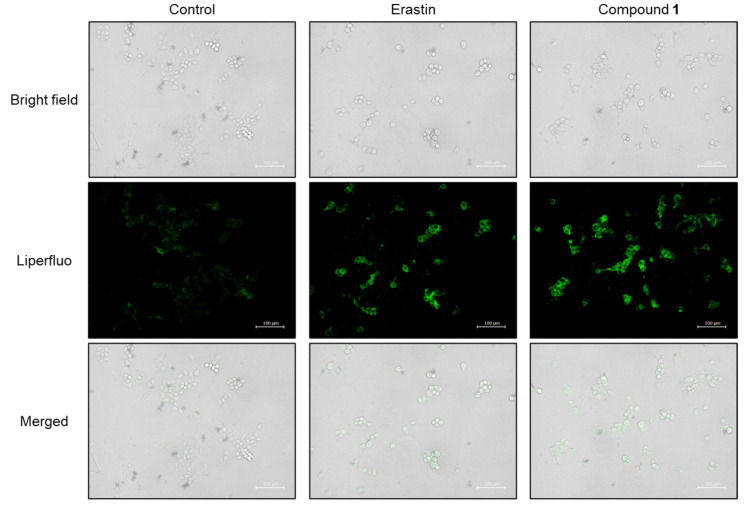
Detection of lipid peroxides in SBC-3 cells. SBC-3 cells were treated with either 0.5 µM of erastin or 2.5 µM of **1** for 24 h. Then, the cells were stained with Liperfluo and observed using a fluorescence microscope. The scale bars indicate 100 μm.

**Figure 12:**
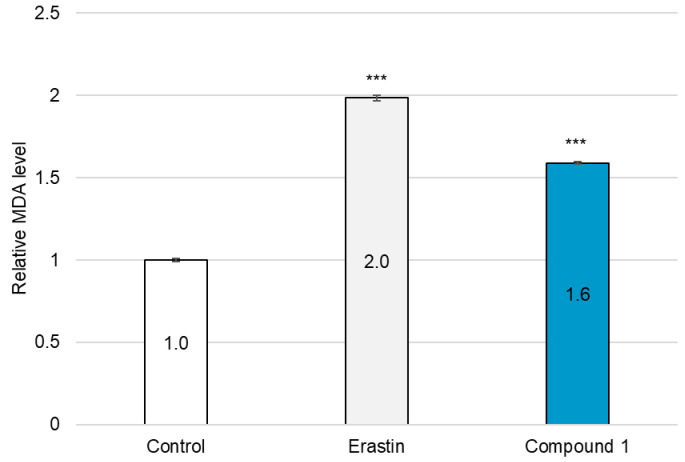
Quantification of MDA in SBC-3 cells. SBC-3 cells were treated with either 0.5 µM of erastin or 2.5 µM of **1** for 24 h. Following treatment, the MDA content in the cells was quantified. The relative MDA levels are presented as the mean ± SEM from experiments performed in triplicate (*** *p* < 0.001 vs. the control).

Hydroxyl radicals are the major initiators of non-enzymatic lipid peroxidation process. Hydroxyl radicals interact with cell membrane polyunsaturated fatty acids (PUFA), leading to the formation of lipid peroxides [32]. Malondialdehyde (MDA) is one of the most abundant and cytotoxic end-products of lipid peroxidation [33]. Since the overproduction of hydroxyl radicals was confirmed in SBC-3 cells treated with **1**, lipid peroxide accumulation was detected, and MDA was quantified. SBC-3 cells were treated with either 0.5 µM of erastin or 2.5 µM of **1**. Following treatment for 24 h, the cells were stained with Liperfluo, a lipid peroxide detection reagent, and observed by fluorescence microscopy (Figure 11). Enhanced bright green fluorescence was observed in cells treated with **1** compared to that in the control, confirmed that lipid peroxides accumulated in SBC-3 cells treated with **1**. Additionally, the MDA generation level was enhanced in the cells treated with **1** (Figure 12).

**Figure 13:**
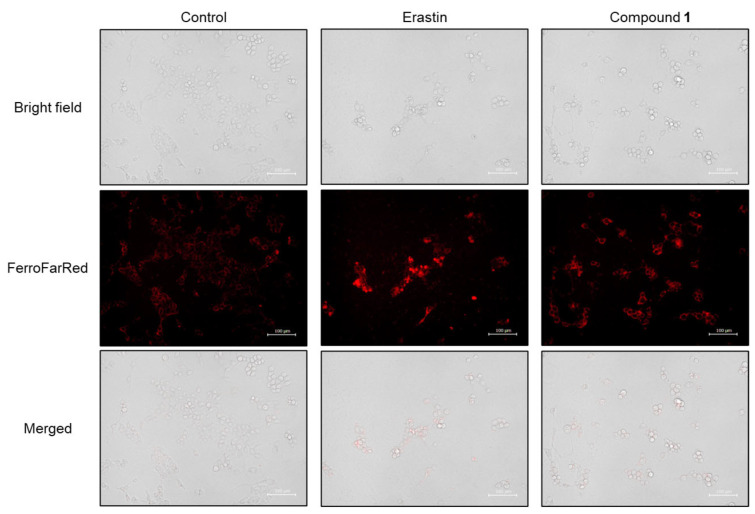
Detection of cellular Fe^2+^ in SBC-3 cells. SBC-3 cells were treated with either 0.5 μM of erastin or 2.5 μM of **1** for 24 h. Then, the cells were stained with FerroFarRed and observed using a fluorescence microscope. The scale bars indicate 100 μm.

Subsequently, the accumulation of cellular Fe^2+^ was detected in SBC-3 cells treated with either 0.5 µM of erastin or 2.5 µM of **1** for 24 h. After treatment, the cells were stained with FerroFarRed, a cellular Fe^2+^ detection reagent, and observed by fluorescence microscopy (Figure 13). Enhanced bright red fluorescence was observed in cells treated with **1** compared to that in the control, confirming that Fe^2+^ was accumulated in SBC-3 cells treated with **1**.

**Figure 14:**
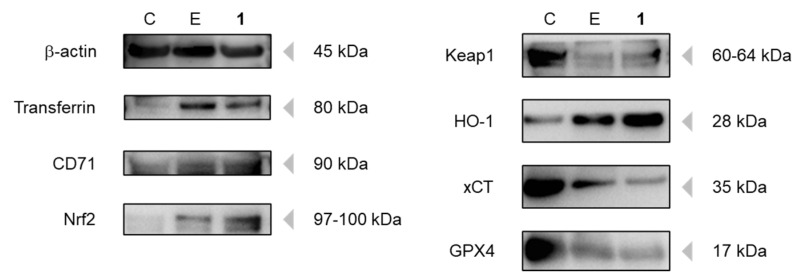
Detection of transferrin, CD71, Nrf2, Keap1, HO-1, xCT, and GPX4 in SBC-3 cells. SBC-3 cells were treated with either 0.5 μM of erastin or 2.5 μM of **1** for 24 h. The extracted proteins of the cells were applied to Western blotting analysis (C: Control; E: Erastin; **1**: Compound **1**).

Since the above data suggested that **1** induced ferroptosis in SBC-3 cells, the expression levels of key ferroptosis-associated proteins were analyzed by Western blot analysis. Protein samples were prepared from SBC-3 cells treated with either 0.5 µM of erastin or 2.5 µM of **1** for 24 h. CD71, also known as transferrin receptor 1, is primarily responsible for cellular iron uptake. When the iron-transferrin complex binds to CD71 on the cell surface, the entire complex is internalized by the cell via receptor-mediated endocytosis [34,35]. When SBC-3 cells were treated with **1**, the expression levels of both transferrin and CD71 were significantly increased, which was consistent with the observed accumulation of intracellular Fe^2+^ (Figure 14). This enhanced iron uptake machinery likely contributes to the ferroptotic cell death.

To maintain cellular homeostasis against oxidative stress, mammalian cells possess the nuclear factor erythroid 2-related factor 2 (Nrf2) Kelch-like ECH-associated protein 1 (Keap1) antioxidant defense system, which is a critical protective mechanism. Under physiological conditions, Nrf2 is sequestered in the cytoplasm by Keap1, which facilitates Nrf2 ubiquitination and subsequent proteasomal degradation, thereby maintaining low basal levels of antioxidant gene expression. When cells encounter oxidative stress, Keap1 undergoes conformational changes that disrupt its interaction with Nrf2, leading to Nrf2 stabilization and nuclear translocation. Upon nuclear entry, Nrf2 binds to the antioxidant response element and activates the transcription of heme oxygenase-1 (HO-1) and other cytoprotective genes, including catalase, NADPH-quinone oxidoreductase-1, and superoxide dismutase [36,37]. Notably, HO-1 catalyzes the degradation of heme to produce biliverdin, carbon monoxide, and free iron (Fe^2+^), which can further contribute to intracellular Fe^2+^ accumulation and potentially enhance ferroptosis [38]. In SBC-3 cells treated with **1**, the expression of Nrf2 and HO-1 was upregulated, whereas that of Keap1 was downregulated (Figure 14). These results indicate that SBC-3 cells exhibited a protective response against the oxidative stress caused by **1**.

xCT, a cystine/glutamate antiporter also known as SLC7A11, plays a crucial role in glutathione synthesis by importing cystine, which is reduced to cysteine for the biosynthesis of reduced glutathione (GSH) [39]. Glutathione peroxidase 4 (GPX4) exhibits phospholipid peroxidase activity and catalyzes the reduction of lipid peroxides, using GSH as a reducing substrate. Therefore, xCT inhibition causes GSH depletion and GPX4 inactivation, leading to the accumulation of lipid peroxides [40]. Erastin specifically inhibits xCT activity by binding to its transporter, thereby inducing glutathione depletion and triggering ferroptosis [40]. Downregulation of both xCT and GPX4 expression was confirmed in SBC-3 cells treated with **1** and erastin (Figure 14).

These findings demonstrate that **1** induces ferroptosis in SBC-3 cells through a dual mechanism involving enhanced cellular iron uptake via upregulation of transferrin and CD71 expression and impaired glutathione synthesis through downregulation of both xCT and GPX4 expression.

## 3. Materials and Methods

### 3.1. General

General materials and instruments for compound isolation, structure identification, cell culture, and cytotoxicity evaluation are provided in the Supplementary Materials.

### 3.2. Plant Material

*A. africanus* was cultivated in the medicinal botanical garden of the Tokyo University of Pharmacy and Life Sciences (TUPLS) (Tokyo, Japan) in November 2017. A voucher specimen has been deposited in the herbarium of the TUPLS.

### 3.3. Extraction and Isolation

The underground parts of *A. africanus* (24 kg, fresh weight) were extracted with MeOH (60 °C, 20 L × 2 times), and the solvent was removed using an evaporator. The extract (910 g) was loaded on a Diaion HP-20 column (*ϕ* 80 mm × 600 mm) and successively eluted with MeOH-H_2_O (3:7), MeOH-H_2_O (1:1), MeOH, EtOH, and EtOAc. The MeOH eluted fraction (fraction C; 160 g) was separated by silica gel CC (*ϕ* 80 mm × 270 mm) eluted with CHCl_3_-MeOH-H_2_O (10:10:1) to obtain three sub-fractions (Frs. C-1–C-3). Fraction C-2 was subjected to silica gel CC eluted with CHCl_3_-MeOH-H_2_O (40:10:1), ODS silica gel CC eluted with MeOH-H_2_O (1:1; 7:3; 3:1) and MeCN-H_2_O (2:3), and preparative ODS HPLC using MeOH-H_2_O (2:1) and MeCN-H_2_O (2:3) to afford **1** (199 mg), **2** (42 mg), and **3** (3.6 mg).

Compound **1**: ^1^H NMR spectral data (500 MHz, pyridine-*d*_5_): δ_H_ 6.30 (d, *J* = 1.1 Hz, H-1″), 4.97 (d, *J* = 7.8 Hz, H-1′′′), 4.84 (d, *J* = 7.6 Hz, H-1′), 1.70 (d, *J* = 6.2 Hz, Me-6″), 1.16 (s, Me-19), 1.10 (d, *J* = 7.0 Hz, Me-21), 0.85 (s, Me-18), 0.67 (d, *J* = 5.6 Hz, Me-27). ^13^C NMR spectral data (125 MHz, pyridine-*d*_5_): δ_C_ 40.0, 70.9, 82.7, 40.3, 73.6, 34.3, 26.5, 34.2, 45.5, 40.5, 21.6, 40.2, 40.9, 56.2, 32.1, 81.2, 63.0, 16.6, 17.2, 41.9, 14.9, 109.2, 31.8, 29.2, 30.5, 66.8, 17.3 (C-1–C-27), 100.8, 77.1, 89.1, 69.6, 77.7, 62.1 (C-1′–C-6′), 102.1, 72.4, 72.7, 74.1, 69.5, 18.5 (C-1″–C-6″), 105.1, 72.3, 75.2, 69.9, 77.4, 62.0 (C-1′′′–C-6′′′). 

Compound **2**: ^1^H NMR spectral data (500 MHz, pyridine-*d*_5_): δ_H_ 6.33 (d, *J* = 1.1 Hz, H-1″), 5.07 (br s, H-7), 4.99 (d, *J* = 7.8 Hz, H-1′′′), 4.87 (d, *J* = 7.4 Hz, H-1′), 1.71 (d, *J* = 6.2 Hz, Me-6″), 1.13 (s, Me-19), 1.11 (d, *J* = 7.0 Hz, Me-21), 0.76 (s, Me-18), 0.69 (d, *J* = 5.7 Hz, Me-27). ^13^C NMR spectral data (125 MHz, pyridine-*d*_5_): δ_C_ 39.8, 70.4, 82.5, 39.6, 73.0, 37.1, 115.9, 139.0, 43.4, 39.8, 21.6, 40.4, 41.6, 55.0, 31.4, 80.9, 62.7, 16.4, 19.0, 42.4, 14.9, 109.2, 31.7, 29.2, 30.5, 66.8, 17.3 (C-1–C-27), 100.8, 77.0, 89.2, 69.5, 77.7, 62.0 (C-1′–C-6′), 102.1, 72.4, 72.7, 74.1, 69.5, 18.5 (C-1″–C-6″), 105.1, 72.3, 75.2, 70.0, 77.4, 62.0 (C-1′′′–C-6′′′).

Compound **3**: ^1^H NMR spectral data (500 MHz, pyridine-*d*_5_): δ_H_ 6.36 (br s, H-1″), 5.73 (br d, *J* = 6.1 Hz, H-11), 5.24 (br d, *J* = 5.1 Hz, H-7), 5.00 (d, *J* = 7.8 Hz, H-1′′′), 4.89 (d, *J* = 7.5 Hz, H-1′), 1.72 (d, *J* = 6.2 Hz, H-6″), 1.28 (s, Me-19), 1.09 (d, *J* = 6.9 Hz, Me-21), 0.78 (s, Me-18), 0.69 (d, *J* = 5.5 Hz, Me-27). ^13^C NMR spectral data (125 MHz, pyridine-*d*_5_): δ_C_ 39.1, 70.7, 82.9, 38.6, 73.0, 37.6, 117.5, 135.6, 142.6, 42.9, 121.0, 42.4, 40.6, 51.7, 31.5, 81.4, 62.2, 15.9, 26.3, 42.6, 14.6, 109.3, 31.7, 29.2, 30.5, 66.9, 17.3 (C-1–C-27), 101.1, 77.0, 89.3, 69.5, 77.7, 62.0 (C-1′–C-6′), 102.1, 72.4, 72.7, 74.1, 69.5, 18.6 (C-1″–C-6″), 105.1, 72.4, 75.3, 70.0, 77.4, 62.0 (C-1′′′–C-6′′′).

### 3.4. Cell Culture and Cytotoxic Activity Assay

SBC-3 cells were grown in minimum essential medium (MEM) supplemented with 10% heat-inactivated fetal bovine serum (FBS), L-glutamine, 100 unit per mL penicillin G sodium salt, and 100 μg per mL streptomycin sulfate, under standard culture conditions (37 °C, 5% CO_2_ in a humidified environment). For the assay, SBC-3 cells were seeded into 96-well flat-bottomed plates (AGC TECHNO GLASS, Shizuoka, Japan) at a density of 2000 cells per well. After the cell adhered to the wall, the MEM was aspirated and replaced with 196 μL of fresh medium. Subsequently, test compounds dissolved in EtOH-H_2_O (1:1) were then added (4 μL per well), and cells were treated for 72 h. Negative control wells received an equivalent volume (4 μL) of the vehicle solution EtOH-H_2_O (1:1). At the end of the treatment period, 10 µL of MTT (DOJINDO, Kumamoto, Japan) solution prepared at 5 mg per mL in PBS was added to each well, followed by a 4 h incubation. The resulting MTT formazan crystals were solubilized in dimethyl sulfoxide (DMSO; FUJIFILM Wako Pure Chemical, Osaka, Japan) prior to absorbance measurement at 550 nm using an SH-1300 Lab microplate reader (CORONA ELECTRIC, Ibaraki, Japan). The half-maximal inhibitory concentration (IC_50_) values for **1**–**3** were determined through analysis of their respective dose-response curves. To assess the cytotoxic effects of **1** on SBC-3 cells after 24 h exposure, the identical protocol was employed, except that the initial seeding density was adjusted to 5000 cells per well.

### 3.5. DAPI Staining

SBC-3 cells were seeded in a 24-well flat-bottomed plate (AGC TECHNO GLASS) at a density of 8.0 × 10^4^ cells per well. After the cells adhered to the wall, the cells were treated with either 6 µM of cisplatin or 2.5 µM of **1** for 24 h. At the end of the treatment period, the plate was centrifuged at 400 *g* for 5 min, followed by aspiration of the supernatant. Cell fixation was performed using 1% glutaraldehyde at 28 °C for 30 min. Subsequent to centrifugation at 400 *g* for 5 min, the cells underwent PBS washing and were subsequently stained with DAPI (DOJINDO) at a concentration of 0.5 µg per mL in PBS at 28 °C for 10 min in darkness. The cells were observed using a BZ-X710 All-in-One Fluorescence Microscope (KEYENCE, Osaka, Japan).

### 3.6. Apoptosis Detection by Annexin V-FITC and PI Double Staining

Apoptotic cell detection was conducted utilizing an Annexin V-FITC Apoptosis Detection Kit (Nacalai-Tesque, Kyoto, Japan), according to the manufacturer’s instructions. SBC-3 cells were seeded in 6-well flat-bottomed plates (AGC TECHNO GLASS) at a density of 1.4 × 10^5^ cells per well. After the cells adhered to the wall, SBC-3 cells were treated with either 6 µM of cisplatin or 2.5 µM of **1** for 24 h. At the end of the treatment period, SBC-3 cells underwent detachment using TrypLE Select (Gibco, Grand Island, NY, USA) and were subsequently washed with PBS for cell pellet collection. The individual cell pellets were resuspended in 1×Annexin V binding buffer and underwent double staining with Annexin V-FITC and PI at 28 °C for 15 min in darkness. Flow cytometry analysis was performed using a BD FACSCelesta flow cytometer (BD Biosciences, Franklin Lakes, NJ, USA).

### 3.7. Analysis of Cell Cycle

SBC-3 cells were seeded in 6-well flat-bottomed plates at a density of 1.4 × 10^5^ cells per well. After the cells adhered to the wall, SBC-3 cells were treated with either 6 μM of cisplatin or 2.5 μM of **1** for 24 h. At the end of the treatment period, the cells underwent detachment using TrypLE Select and were subsequently washed with PBS for cell pellet collection. Subsequently, the individual cell pellets were subjected to fixation with EtOH-PBS (7:3) at −20 °C overnight. After centrifugation, the supernatant was aspirated, and the cells were stained using FxCycle PI/RNase Staining Solution (Thermo Fisher Scientific, Waltham, MA, USA) at 28 °C for 15 min in darkness. Flow cytometry analysis was performed using a BD FACSCelesta flow cytometer (BD Biosciences).

### 3.8. Detection of Activated Caspases

SBC-3 cells were harvested in 6-well flat-bottomed plates at a density of 1.4 × 10^5^ cells per well. After the cells adhered to the wall, SBC-3 cells were exposed to either 6 μM of cisplatin or 2.5 μM of **1** for 24 h. Upon completion of the incubation period, the cells underwent detachment using TrypLE Select and were subsequently subjected to PBS washing for pellet preparation. Then, the individual cell pellets underwent fixation with a 4% paraformaldehyde solution at 28 °C for 15 min. After fixation, the cells were rinsed with 1% bovine serum albumin (BSA; Nacalai-Tesque)-containing PBS and centrifuged at 15,000 rpm for 5 min. The cell pellets were subsequently resuspended in MeOH-1% BSA-containing PBS (9:1) and kept for 30 min on ice. Following centrifugation and supernatant removal, the cells were rinsed with 1% BSA-containing PBS. The cells were subsequently incubated with a primary antibody solution for 20 min on ice, followed by washing with 1% BSA-containing PBS. Subsequently, the cells were incubated with a secondary antibody solution for 20 min on ice in darkness and rinsed again with 1% BSA-containing PBS. Finally, the cell pellets were resuspended in 1% BSA-containing PBS and subjected to analysis using a BD FACSCelesta flow cytometer (BD Biosciences). The following primary antibodies were used: Cleaved caspase-3 (Asp 175) (5A1E, Rabbit mAb, #9664, 1:1000; Cell Signaling Technology, Danvers, MA, USA), Cleaved caspase-8 (Asp 374) (18C8, Rabbit mAb, #9496, 1:500; Cell Signaling Technology), Cleaved caspase-9 (Asp 315) (D8I9E, Rabbit mAb, #20750, 1:200; Cell Signaling Technology). The following secondary antibody was used: Anti-rabbit IgG (H + L), F(ab’)_2_ Fragment (Alexa Fluor 488 conjugate) (#4412, 1:500; Cell Signaling Technology).

### 3.9. Detection of Mitochondrial Membrane Potential

Mitochondrial membrane potential assessment was conducted utilizing the JC-1 MitoMP Detection Kit (DOJINDO), in accordance with the manufacturer’s instructions. SBC-3 cells were harvested in 6-well flat-bottomed plates at a density of 1.4 × 10^5^ cells per well. After the cells adhered to the wall, SBC-3 cells were exposed to either 6 μM of cisplatin or 2.5 μM of **1** for 24 h. Upon completion of the incubation period, the cells were detached using TrypLE Select and subsequently subjected to PBS washing for pellet preparation. Cell staining was carried out with 4 μM of JC-1 for 45 min in a CO_2_ incubator. Flow cytometry analysis was performed using a BD FACSCelesta flow cytometer (BD Biosciences).

### 3.10. Detection of ROS Generation Levels

ROS generation level assessment was performed utilizing the CellROX Green Flow Cytometer Assay Kit (Thermo Fisher Scientific), in accordance with the manufacturer’s instructions. SBC-3 cells were seeded in 6-well flat-bottomed plates at a density of 1.4 × 10^5^ cells per well. After the cells adhered to the wall, SBC-3 cells were treated with either 6 μM of cisplatin or 2.5 μM of **1** for 24 h. At the end of the treatment period, the cells underwent detachment using TrypLE Select and were subsequently washed with PBS for cell pellet collection. Cell staining was conducted using 750 nM of CellROX Green for 1 h in a CO_2_ incubator. Flow cytometry analysis was performed using a BD FACSCelesta flow cytometer (BD Biosciences).

### 3.11. Detection of Hydroxyl Radicals

Hydroxyl radical detection was performed utilizing the HPF reagent (Goryo Chemical, Hokkaido, Japan), according to the manufacturer’s instructions. SBC-3 cells were seeded in a 24-well flat-bottomed plate at a density of 5.8 × 10^4^ cells per well. After the cells adhered to the wall, SBC-3 cells were treated with either 0.5 μM of erastin or 2.5 μM of **1** for 24 h. At the end of the treatment period, the plate was centrifuged at 1500 rpm for 5 min, followed by supernatant aspiration. Cell staining was conducted using 5 μM of HPF for 30 min in a CO_2_ incubator. Subsequently, the plate was centrifuged at 1500 rpm for 5 min, and the supernatant was removed. Finally, each well received Hank’s balanced salt solution (HBSS; Gibco), and the cells were observed using a BZ-X710 All-in-One Fluorescence Microscope (KEYENCE).

### 3.12. Detection of Lipid Peroxides

Lipid peroxide detection was performed utilizing the Liperfluo reagent (DOJINDO), according to the manufacturer’s instructions. SBC-3 cells were seeded in a 24-well flat-bottomed plate at a density of 5.8 × 10^4^ cells per well. After the cells adhered to the wall, SBC-3 cells were treated with either 0.5 μM of erastin or 2.5 μM of **1** for 24 h. At the end of the treatment period, the plate was centrifuged at 1500 rpm for 5 min, followed by supernatant aspiration. Cell staining was conducted using 10 μM of Liperfluo in a CO_2_ incubator. Following a 1 h incubation period, the plate was centrifuged at 1500 rpm for 5 min, and the supernatant was removed. Finally, each well received PBS, and the cells were observed using a BZ-X710 All-in-One Fluorescence Microscope (KEYENCE).

### 3.13. Quantification of Malondialhyde

Malondialdehyde quantification was performed utilizing the MDA Assay Kit (DOJINDO), in accordance with the manufacturer’s instructions. SBC-3 cells were seeded in 100 mm^2^ dishes at a density of 1.0 × 10^6^ cells per dish. After the cells adhered to the wall, SBC-3 cells were treated with either 0.5 μM of erastin or 2.5 μM of **1** for 24 h. At the end of the treatment period, the cells underwent detachment using TrypLE Select and were subsequently washed with PBS for cell pellet collection. The individual cell pellets were suspended in lysis buffer and maintained at 28 °C for 5 min. Then, the cells were reconstituted in the working solution and subjected to heating at 95 °C for 15 min. Finally, the cells were centrifuged at 10,000× *g* for 10 min, and the supernatant was transferred to a 96-well black-plate (Greiner Bio-One GmbH, Kremsmünster, Austria). Fluorescence measurement was conducted using a Nivo multimode plate reader Alpha S (Revvity, Waltham, MA, USA).

### 3.14. Detection of Cellular Fe^2+^

Cellular Fe^2+^ detection was performed utilizing the FerroFarRed reagent (Goryo Chemical), according to the manufacturer’s instructions. SBC-3 cells were seeded in a 24-well flat-bottomed plate at a density of 5.8 × 10^4^ cells per well. After the cells adhered to the wall, SBC-3 cells were treated with either 0.5 μM of erastin or 2.5 μM of **1** for 24 h. At the end of the treatment period, the plate was centrifuged at 1500 rpm for 5 min, followed by supernatant aspiration. Cell staining was conducted using 10 μM of FerroFarRed in a CO_2_ incubator. Following a 1 h period, the plate was centrifuged at 1500 rpm for 5 min, and the supernatant was removed. Finally, each well received HBSS, and the cells were observed using a BZ-X710 All-in-One Fluorescence Microscope (KEYENCE).

### 3.15. Detection of Ferroptosis-Associated Proteins

SBC-3 cells were seeded in 6-well flat-bottomed plates at a density of 1.4 × 10^5^ cells per well. After the cells adhered to the wall, SBC-3 cells were treated with either 0.5 μM of erastin or 2.5 μM of **1** for 24 h. At the end of the treatment period, the cells underwent detachment using TrypLE Select and were subsequently washed with PBS for cell pellet collection. The individual cell pellets were suspended in RIPA buffer (FUJIFILM Wako Pure Chemical) supplemented with Halt Protease and Phosphatase Inhibitor Cocktail, EDTA-free (100×) (Thermo Fisher Scientific) and kept on ice for 30 min to extract cellular proteins. The cells were subsequently centrifuged at 10,000 *g* for 30 min, and the supernatant containing protein was collected. Protein quantification was performed utilizing the TaKaRa BCA Protein Assay Kit (Takara Bio, Shiga, Japan). Following quantification, the sample protein solutions were loaded onto NuPAGE 4–12% Bis-Tris Gel (Thermo Fisher Scientific), and electrophoresis was conducted employing a WSE-1165 RapidasMinislab electrophoresis tank (ATTO, Tokyo, Japan). Protein transfer from the gel to a polyvinylidene difluoride (PVDF) membrane was accomplished using a Protein Transfer Kit for Semidry (COSMO BIO, Tokyo, Japan) and a WSE 4025HorizeBLOT 2M blotting device (ATTO). Following transfer, the PVDF membrane was agitated in a Blocking One solution (Nacalai-Tesque) at 28 °C for 30 min. The PVDF membrane was subjected to incubation with the following primary antibodies dissolved in Can Get Signal Immunoreaction Enhancer Solution 1 (TOYOBO, Osaka, Japan) at 4 °C overnight duration: β-actin (8H10D10 Mouse mAb, #3700, 1:1000; Cell Signaling Technology), Nrf2 (D1Z9C XP Rabbit mAb, #12721, 1:1000; Cell Signaling Technology), Keap1 (D6B12, Rabbit mAb, #8047, 1:1000; Cell Signaling Technology), HO-1 (E3F4S Rabbit mAb, #43966, 1:1000; Cell Signaling Technology), xCT (D2M7A Rabbit mAb, #12691, 1:1000; Cell Signaling Technology), Transferrin (E7F4T Rabbit mAb, #35293, 1:1000, Cell Signaling Technology), CD71 (D7G9X XP Rabbit mAb, #13113, 1:1000; Cell Signaling Technology), and GPX4 (EPNCIR144, ab125066 Rabbit mAb, 1:1000; abcam, Cambridge, UK). The PVDF membrane underwent agitation in TBS-T buffer (1×), and was subsequently treated with secondary antibodies (Anti-mouse IgG, HRP linked Antibody, #7076, 1:10,000 or Anti-rabbit IgG, HRP linked Antibody, #7074, 1:10,000; Cell Signaling Technology) dissolved in Can Get Signal Immunoreaction Enhancer Solution 2 (TOYOBO) at 28 °C for 1 h. Finally, the PVDF membrane underwent incubation with ECL Prime Western Blotting Detection Reagents (GE Healthcare, Boston, MA, USA), and detection was conducted using a LAS-3000 luminescent image analyzer (FUJIFILM, Tokyo, Japan).

### 3.16. Statistical Analysis

Statistical analysis was performed by a one-way analysis of variance (ANOVA) followed by Dunnett’s test. A probability (*p*) value of less than 0.001 was considered to indicate a statistically significant difference.

## 4. Conclusions

**Figure 15:**
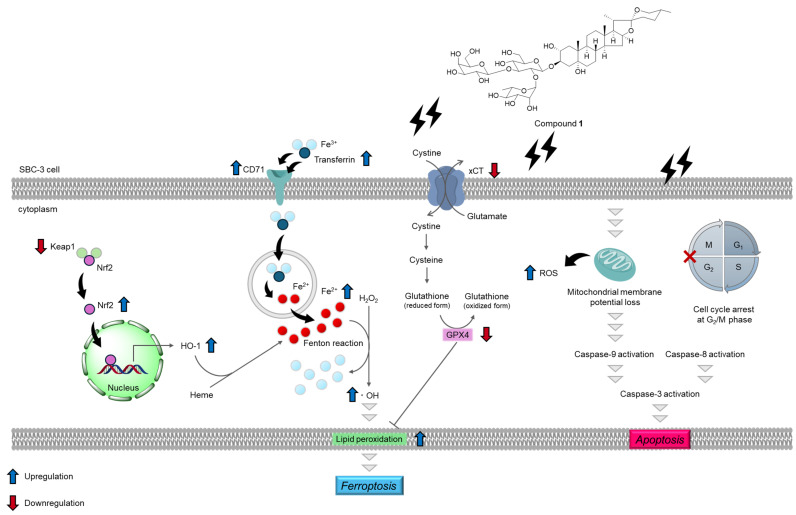
Summary of apoptosis- and ferroptosis-inducing activities of **1**. Compound **1** induces apoptosis in SBC-3 cells via the mitochondrial pathway, involving cell cycle arrest at the G_2_/M phase in SBC-3 cells. Additionally, **1** also induces ferroptosis in SBC-3 cells, accompanied by upregulation of transferrin, CD71, Nrf2, and HO-1 expression; downregulation of Keap1, xCT, and GPX4 expression; and accumulation of cellular Fe^2+^, hydroxyl radicals, and lipid peroxides.

Three spirostan-type steroidal glycosides (**1**–**3**) were isolated from the underground parts of *A. africanus*. Compounds **1**–**3** exhibited cytotoxicity against SBC-3 cells, a representative human SCLC cell line, with IC_50_ values of 0.56, 1.4, and 7.4 μM, respectively. Compound **1**, which showed the most potent cytotoxicity among the isolated compounds, arrested the cell cycle of SBC-3 cells at the G_2_/M phase and induced apoptosis primarily via the mitochondrial pathway, characterized by the activation of caspases-3 and -9, mitochondrial membrane potential loss, and overproduction of ROS. Furthermore, **1** induced ferroptosis through a dual mechanism involving enhanced cellular iron uptake via upregulation of transferrin and CD71 expression and impaired glutathione synthesis through downregulation of both xCT and GPX4 expression (Figure 15). Compound **1** induced cell death through both apoptotic and ferroptotic pathways, suggesting its potential as a seed compound for anticancer drug development against SCLC.

## Data Availability

Data are contained within the article.

## Supplementary Materials

The following supporting information can be downloaded at: https://www.mdpi.com/article/10.3390/molecules30153189/s1, Figure S1: ^1^H NMR spectrum of **1**; Figure S2: ^13^C NMR spectrum of **1**; Figure S3: ^1^H NMR spectrum of **2**; Figure S4: ^13^C NMR spectrum of **2**; Figure S5: ^1^H NMR spectrum of **3**; Figure S6: ^13^C NMR spectrum of **3**.
