# Supplementary material for: Agapanthussaponin A from the Underground Parts of Agapanthus africanus Induces Apoptosis and Ferroptosis in Human Small-Cell Lung Cancer Cells
Source: Molecules. 2025 Jul 30;30(15):3189. doi: 10.3390/molecules30153189 (PMC12348243; doi:10.3390/molecules30153189)
Supplement: Supplementary file 1 [file molecules-30-03189-s001.zip › molecules-3749974-supplementary.pdf]

-Supplementary materials-

Agapanthussaponin A from the underground parts of *Agapanthus africanus* induces apoptosis and ferroptosis in human small-cell lung cancer cells

Tomoki Iguchi [1,*], Tamami Shimazaki [1], and Yoshihiro Mimaki [1]

[1]Department of Medicinal Pharmacognosy, School of Pharmacy, Tokyo University of Pharmacy and Life Sciences, 1432-1, Horinouchi, Hachioji, Tokyo, 192-0392, Japan; y184116@toyaku.ac.jp (T.S.), mimakiy@toyaku.ac.jp (Y.M.)

Correspondence: iguchit@toyaku.ac.jp; Tel.: +81-42-676-4575

Table of Contents

- General experimental procedures
- Figure S1. ^{1}H NMR spectrum of **1**
- Figure S2. ^{13}C NMR spectrum of **1**
- Figure S3. ^{1}H NMR spectrum of **2**
- Figure S4. ^{13}C NMR spectrum of **2**
- Figure S5. ^{1}H NMR spectrum of **3**
- Figure S6. ^{13}C NMR spectrum of **3**

General experimental procedures

Measurement of NMR spectra were performed using an AVANCE III HD-500 (500 MHz for ^{1}H NMR; 125 MHz for ^{13}C NMR) spectrometer (Bruker, Billerica, MA, USA) at 300 K, and chemical shifts were displayed as δ values (ppm). Diaion HP-20 porous polymer polystyrene resin (Mitsubishi-Chemical, Tokyo, Japan), silica gel Chromatorex BW-300 (Fuji-Silysia Chemical, Aichi, Japan), and ODS silica gel COSMOSIL $75C_{18}$-OPN (Nacalai-Tesque, Kyoto, Japan) were used as CC packings. Precoated silica gel $60F_{254}$ or RP18 $F_{254}S$ plates (0.25 mm thick, Merck, Darmstadt, Germany) were applied for thin-layer chromatography (TLC) analysis. The spots were visualized by spraying the developed TLC plate with H_2SO_4-H_2O (1:9) followed by heating. The preparative high-pressure liquid chromatography (HPLC) system consisted of an LC-20AD pump (Shimadzu, Kyoto, Japan), an RID-10A detector (Shimadzu), a TSKgel ODS-100Z column (10 mm i.d. × 250 mm, 5 μm; Tosoh, Tokyo, Japan), and a Rheodyne injection port (Thermo Fisher Scientific, Waltham, MA, USA). Cell culture and cytotoxicity assay were conducted using the following materials and reagents: minimum essential medium (MEM), 0.25 % trypsin-ethylenediaminetetraacetic acid (EDTA) solution, and cisplatin (Sigma, St. Louis, MO, USA); penicillin G sodium salt and streptomycin sulfate (Gibco, Gland Island, NY, USA); fetal bovine serum (FBS; NICHIREI BIOSCIENCES, Tokyo, Japan); SBC-3 cells (JCRB0818; Japanese Collection of Research Bioresources Cell Bank; National Institute of Biomedical Innovation, Health and Nutrition, Osaka, Japan); phosphate-buffer saline (PBS) (FUJIFILM Wako Pure Chemical, Osaka, Japan); Countess II FL automated cell counter (Thermo Fisher Scientific); MCO-170AIC-PJ CO_2 incubator (PHC, Tokyo, Japan).

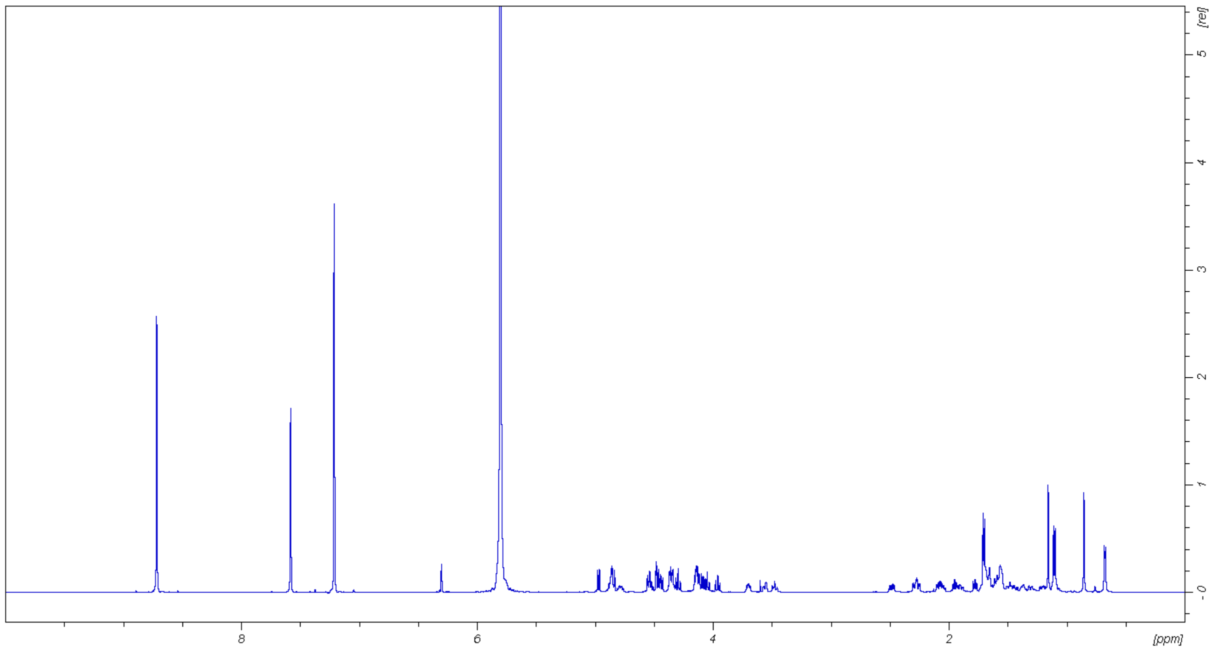

Figure S1. ^{1}H NMR spectrum of **1**

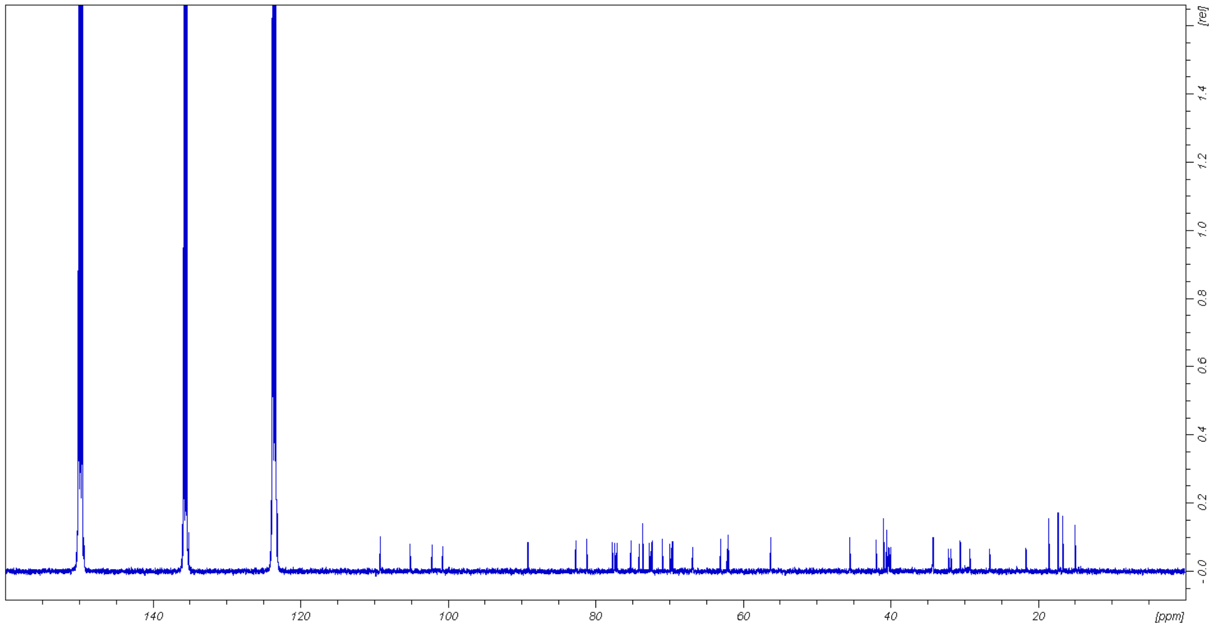

Figure S2. ^{13}C NMR spectrum of **1**

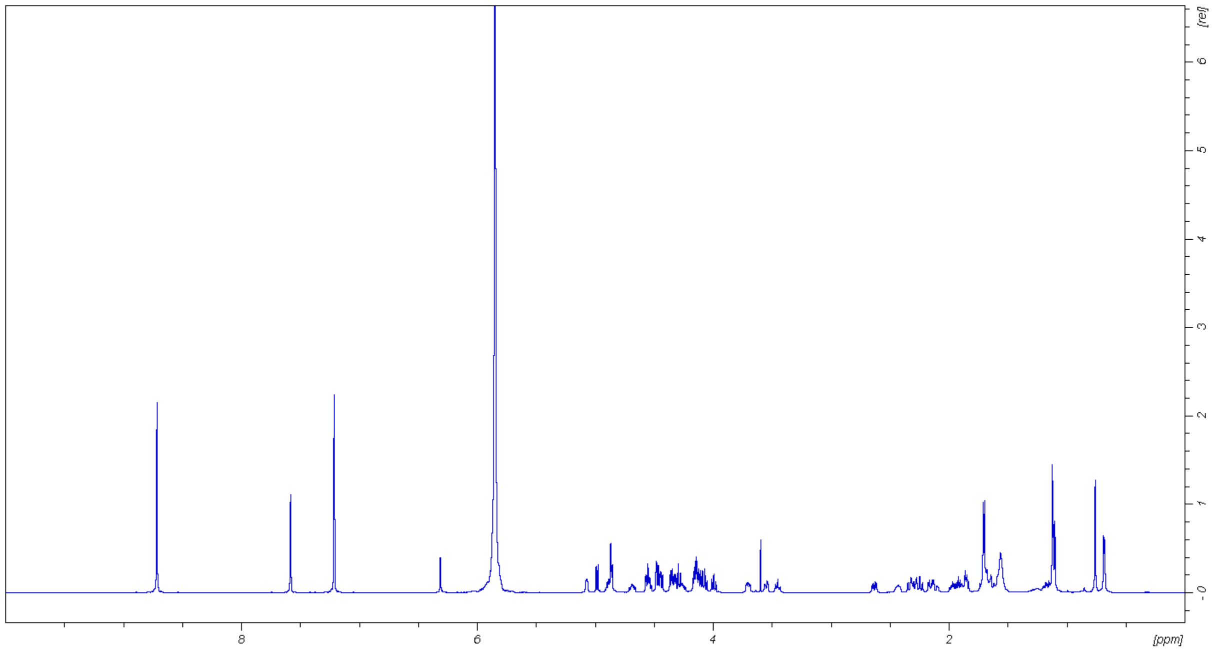

Figure S3. ^{1}H NMR spectrum of **2**

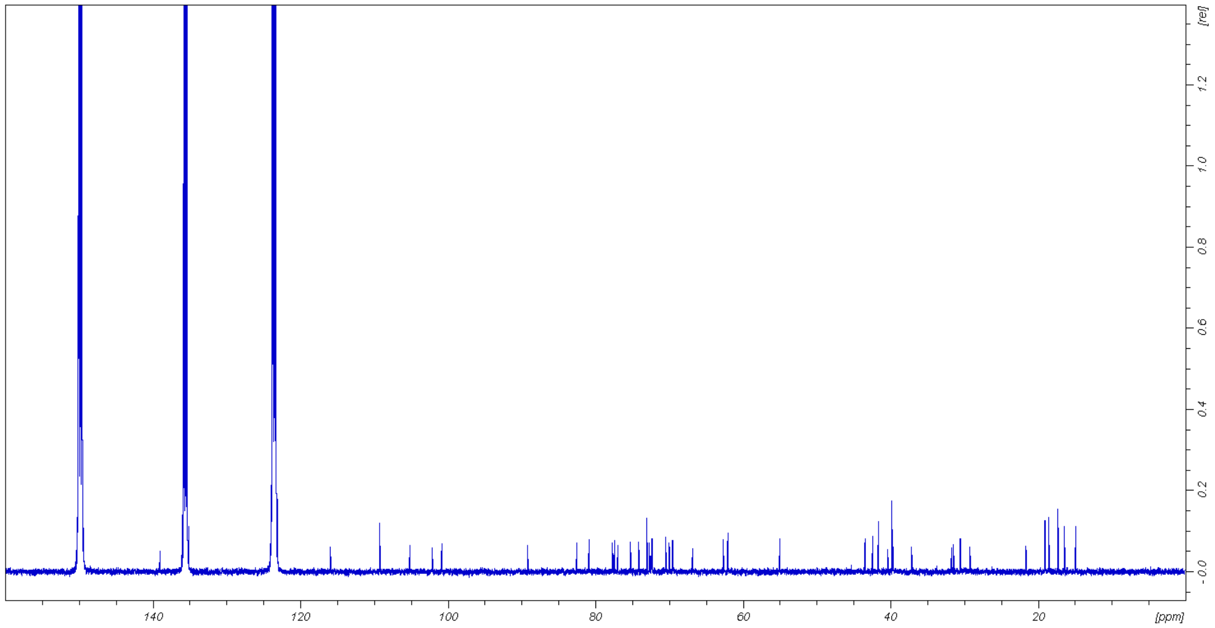

Figure S4. ^{13}C NMR spectrum of **2**

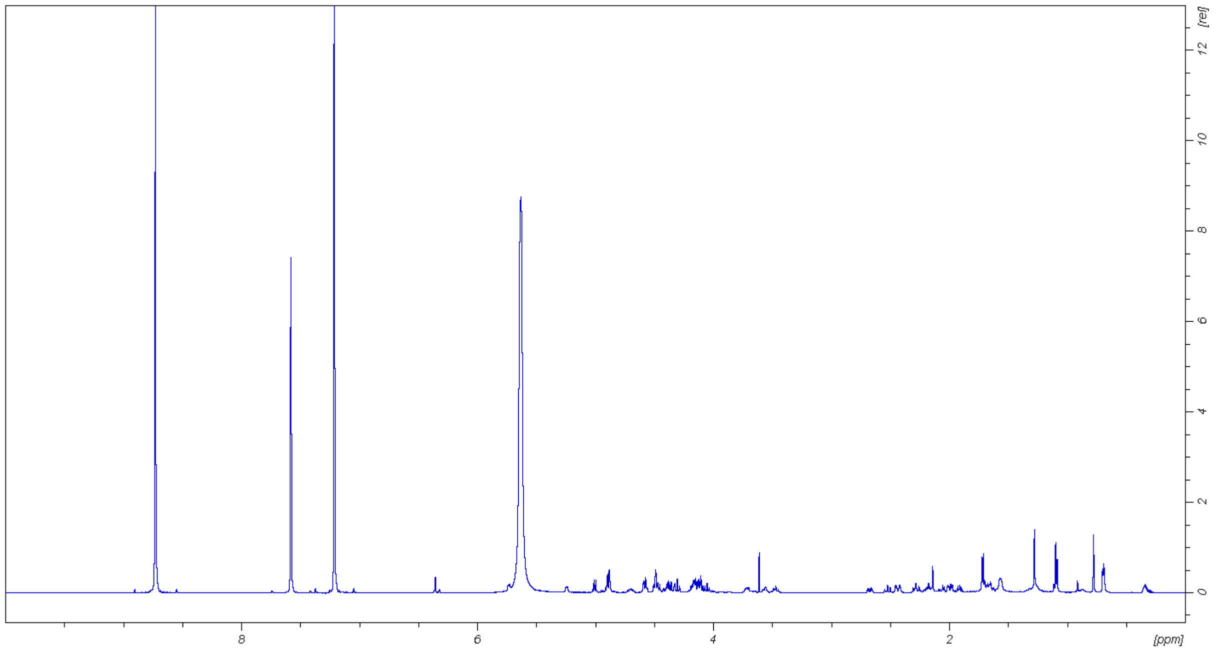

Figure S5. ^{1}H NMR spectrum of **3**

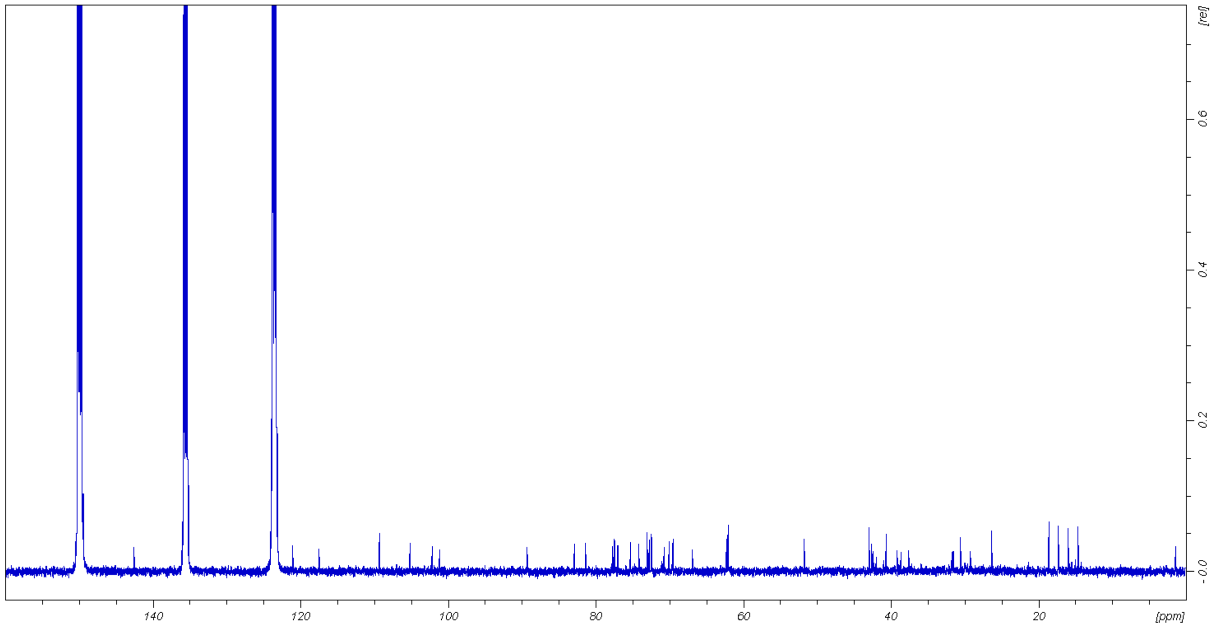

Figure S6. ^{13}C NMR spectrum of **3**
